# Evidence-informed capacity building for setting health priorities in low- and middle-income countries: A framework and recommendations for further research

**DOI:** 10.12688/f1000research.10966.1

**Published:** 2017-03-07

**Authors:** Ryan Li, Francis Ruiz, Anthony J Culyer, Kalipso Chalkidou, Karen J Hofman

**Affiliations:** 1 Global Health and Development Group, Institute of Global Health Innovation, Imperial College London, London, UK; 2 University of York, York, UK; 3 Institute of Health Policy, Management and Evaluation, University of Toronto, Toronto, Ontario, Canada; 4 Priority Cost Effective Lessons for System Strengthening South Africa (PRICELESS SA), MRC/Wits Rural Public Health and Health Transitions Research Unit, Wits University School of Public Health, Johannesburg, South Africa

**Keywords:** health technology assessment, evidence-informed priority setting, health policy, institutions, universal health coverage, knowledge transfer and exchange, capacity development, INNE framework

## Abstract

Priority-setting in health is risky and challenging, particularly in resource-constrained settings. It is not simply a narrow technical exercise, and involves the mobilisation of a wide range of capacities among stakeholders – not only the technical capacity to “do” research in economic evaluations. Using the Individuals, Nodes, Networks and Environment (INNE) framework, we identify those stakeholders, whose capacity needs will vary along the evidence-to-policy continuum. Policymakers and healthcare managers require the capacity to commission and use relevant evidence (including evidence of clinical and cost-effectiveness, and of social values); academics need to understand and respond to decision-makers’ needs to produce relevant research. The health system at all levels will need institutional capacity building to incentivise routine generation and use of evidence. Knowledge brokers, including priority-setting agencies (such as England’s National Institute for Health and Care Excellence, and Health Interventions and Technology Assessment Program, Thailand) and the media can play an important role in facilitating engagement and knowledge transfer between the various actors. Especially at the outset but at every step, it is critical that patients and the public understand that trade-offs are inherent in priority-setting, and careful efforts should be made to engage them, and to hear their views throughout the process. There is thus no single approach to capacity building; rather a spectrum of activities that recognises the roles and skills of all stakeholders. A range of methods, including formal and informal training, networking and engagement, and support through collaboration on projects, should be flexibly employed (and tailored to specific needs of each country) to support institutionalisation of evidence-informed priority-setting. Finally, capacity building should be a two-way process; those who build capacity should also attend to their own capacity development in order to sustain and improve impact.

## Introduction

Setting priorities in health is demanding, risky and fraught with fearsome challenges. One can be caught out by getting them right, for instance when an influential person sees them as a threat to their interests; and one can be caught out by getting them wrong, which often results in the country’s resources being wasted by not having the biggest impact possible on people’s health.

The international Decision Support Initiative (iDSI, www.idsihealth.org) is a practitioner-led partnership that facilitates priority-setting ( Chalkidou *et al.*, 2016b; Li *et al.*, 2016). Its mission is to guide decision-makers towards effective and efficient healthcare resource allocation strategies for improving people’s health. It aims to achieve this by providing a combination of practical support (hands-on technical assistance and institutional strengthening) ( Glassman *et al.*, 2012) and knowledge products (high-quality, policy relevant research and tools).

As part of iDSI’s inception phase in 2014-15, iDSI committed to scoping out an ‘evidence-informed capacity building programme’ that sheds light on the capacity gaps in low- and middle-income countries (LMIC) when it comes to setting health priorities, and explored how they would begin to address these. The programme included an in-depth review of priority-setting capacity in Sub-Saharan Africa ( Doherty, 2015). This paper draws on that review and on broader literature and frameworks concerning capacity building, in an attempt to provide some generalizable insights that could be applied by iDSI in future, and indeed by other stakeholders for priority-setting in LMICs.

Capacity for setting health priorities can be addressed at different levels. Within the broader health policy and political environment, this means examining the central agencies and governmental structures that direct and govern the system and their capacity to deliver whatever has been determined to be their tasks in priority-setting. It also means ensuring that there is effective communication and control that makes the system a functioning network rather than just an assembly of unconnected parts. At the organisational and individual levels, one must address the capacities of specific players or stakeholders in the system and whether these fulfil their purposes. The goal of this capacity building should include transitioning from resource allocation strategies that are historically based on disease burden, “expert opinion” or global advocacy ( Chalkidou *et al.*, 2016b). A more strategic approach to priority-setting would be informed by evidence (that is, evidence on cost-effectiveness and social values as well as disease burden) and deliberative processes ( Balthussen *et al.*, 2016; Chalkidou *et al.*, 2016a; Chalkidou *et al.*, 2016b; Culyer & Lomas, 2006; Lomas *et al.*, 2005).

## Aims, objectives, and scope

In this paper we outline the kinds of capacity needed to support decision makers when setting health priorities, where such capacity can be found, and how best it can be created. We set out a framework for understanding the key elements of capacity building, how iDSI partners are currently involved in supporting capacity development, and finally a research and action agenda that seeks to inform any future capacity building strategy, adopted by iDSI or other development initiatives. We do not provide an exhaustive map of all possible stakeholders and solutions in priority-setting, but offer a starting point for thinking about who the most important stakeholders are and how best they might be approached.

## A framework for understanding capacity building

**Figure 1.**
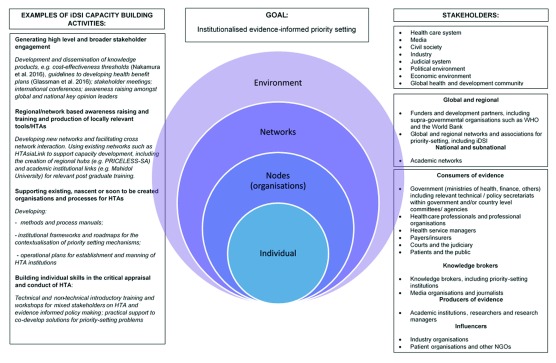
INNE framework applied to iDSI stakeholders and activities.

The United Nations Development Programme INNE Model is one way in which thinking about capacity can be organised ( UNESCO International Institute for capacity Building in Africa, 2006). This model covers four general categories of capacity building: Individual, Node, Network and Enabling Environment, each of which has distinctive characteristics and require different approaches to building capacity further, especially to deliver what is required for universal health coverage (UHC). Each category also entails different segments of the population, whom we conventionally term ‘stakeholders’ ( Thaiprayoon & Smith, 2015; UNESCO International Institute for capacity Building in Africa 2006). Figure 1 gives examples of how existing and future planned activities of the iDSI partnership fit within the INNE framework.

**Figure 2.**
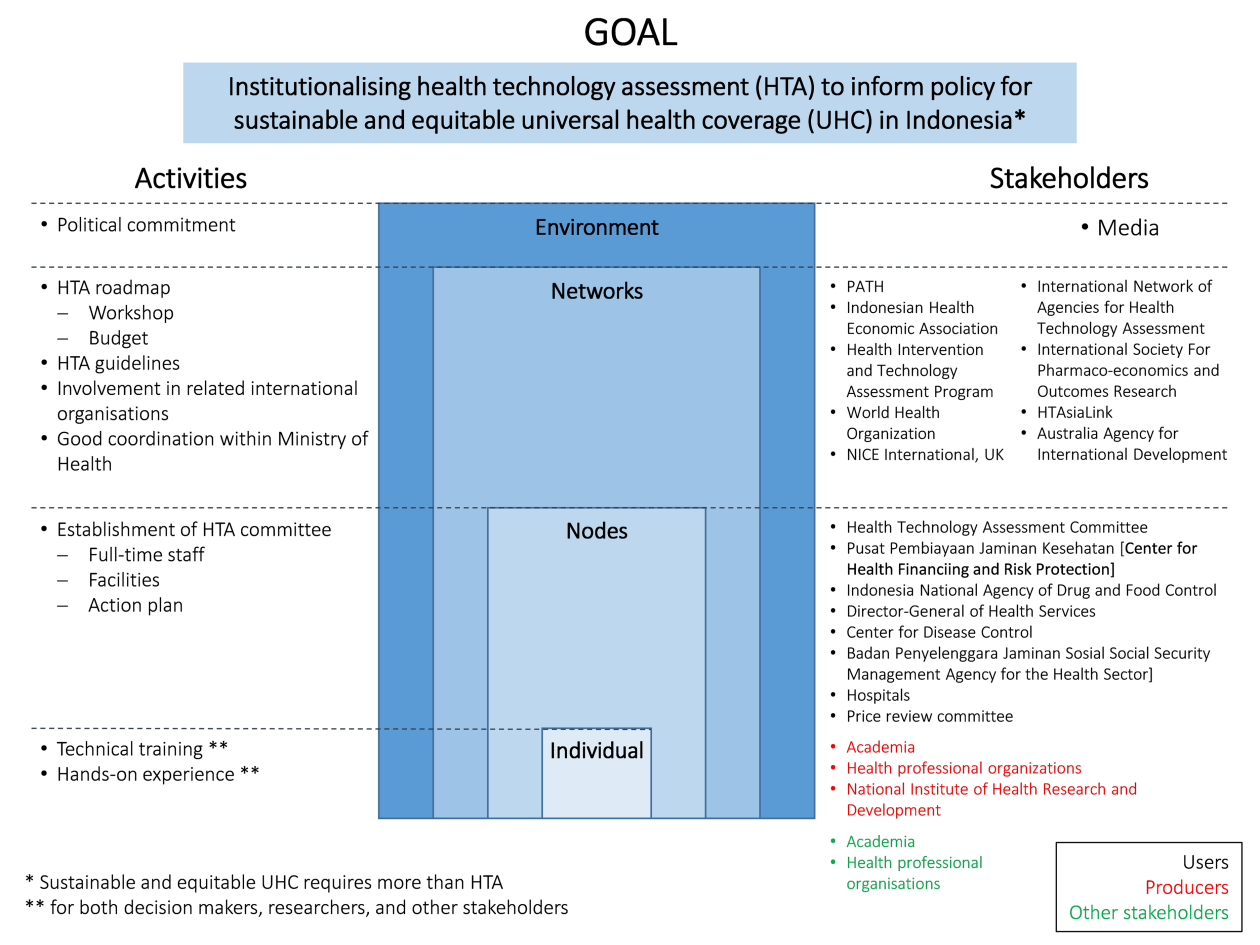
Mapping of institutional stakeholders of health priority-setting in Indonesia, using the INNE framework (adapted from HITAP International Unit, 2015).

iDSI’s practical support in Indonesia provides an example of how the INNE framework can be applied to inform capacity building in health technology assessment (HTA, Figure 2) ( HITAP International Unit, 2015). During a HTA workshop for policymakers and researchers in priority-setting, participants identified relevant stakeholders and populated the framework with activities that would enable Indonesia to reach the end goal of institutionalising HTA for sustainable and equitable UHC.

The iDSI Reference Case for Economic Evaluation, which details principles, methods and reporting standards for the planning and conduct of economic evaluation, has a specific focus on LMIC decision-makers ( Wilkinson *et al.*, 2016) and is an intervention at the Environmental level of INNE. Its preparation involved high-level, global stakeholder engagement ranging from the Bill and Melinda Gates Foundation, who initially commissioned the work, to researchers and policymakers from LMICs as well as high-income countries.

Capacity building activities at one level within the INNE framework can have an impact on, and be influenced by, interventions at other levels ( UNESCO International Institute for capacity Building in Africa, 2006). For example, the development of regional HTA ‘hubs’ is an important aspect of the iDSI approach to capacity building, which is as an intervention at the Network level of the INNE. The two country hubs, one in South Africa (Priority Cost Effective Lessons for System Strengthening, PRICELESS-SA) and another in China (China National Health and Development Research Center), are focal points for networks of academic institutions and government-aligned think tanks, aimed at eventually supporting neighbouring countries in using evidence in policymaking ( Hofman *et al.*, 2015; Zhao, 2016) in areas such as health benefit package design or updating formularies ( Li *et al.*, 2016). The creation of these regional hubs will always involve the strengthening of existing or nascent processes and methods for HTA within the hub countries themselves – in other words, capacity building at the Node level of the INNE framework ( Figure 1).

Local and regional capacity strengthening can occur in parallel, through collaboration between institutions on specific projects. An example is the ongoing collaboration between PRICELESS-SA at the University of the Witwatersrand with the University of KwaZulu Natal to support the refinement of the Essential Medicines List in Tanzania. This technical assistance project not only provides a service to the client country, Tanzania, but it supports the hub’s own capacity development and helps establish the relationships needed to support HTA use and development within South Africa and the region ( Hofman *et al.*, 2015).

The framework makes it clear that, in capacity building, there is a broad range of stakeholder groups to be targeted at country, regional and global levels. Some of these groups operate across INNE levels. For instance, the National Institute for Health and Care Excellent (NICE) in the UK and Health Intervention and Technology Assessment Program (HITAP) in Thailand can be thought of as ‘knowledge brokers’, whose core function is to support the translation of evidence into policy in priority-setting, through convening and interfacing between researchers and decision-makers ( Jongudomsuk *et al.*, 2012; Lomas, 2007). Thus NICE and HITAP are a special example of Nodes that have significant functions across the Network of academic, clinical and policy institutions.

It also follows that there is no single approach to capacity building to support effective priority-setting, but rather a spectrum of activities that identifies the different roles and skill sets of all involved in the process. Focusing on narrowly defined ‘technical’ or research-related activities will not address the reality that priority setting in health takes place within a broader institutional and political framework ( Hawkins & Parkhurst, 2016). This reinforces the value of viewing capacity within the INNE framework and of adopting a tailored approach to building it that addresses the different needs of actors within the system. However, it does require identification and categorisation of all relevant stakeholders. We therefore recommend that a tool for mapping stakeholder groups be developed that can be adapted to the context of different countries.

## Types of capacity

Table 1 lists the principal target stakeholders and capacity needs. It is not intended to be an exhaustive list, but rather a starting point for clarifying the types of capacity that may have to be built and their related activities.

**Table 1.** Different stakeholders in priority-setting require a range of capacities to generate and use evidence and institutionalise good practice into routine.

Stakeholder group	Capacities required
*Environment*	
*Health system*	• To support the capacities required at the different INNE levels by institutionalising evidence-informed priority- setting agencies at provincial, national and regional levels (as deemed appropriate). This perhaps can be seen as one of the goals for the capacity-building activities. Other activities or interventions at the health system level may help or hinder the development and uptake of evidence.
*Networks*	
*Funders and* *development* *partners*	• To commission, receive, interpret and use (as they judge to be appropriate) the methods and outcomes of HTA/ priority-setting research to inform decisions about both investment choices in global health and single technology or program choices at a national level, including investments to support effective priority-setting and health system strengthening. • To have common understanding, for instance through a common theory of change, of the outcomes, preconditions, underlying assumptions of investments to support priority-setting and health system strengthening; and to support knowledge translation efforts towards those outcomes.
*Nodes (organisations) and Individuals*	
*Consumers of evidence*	
*Policy and* *professional* *decision-makers*	• To commission, receive, interpret and use (as they appropriate) the methods and outcomes of HTA and priority- setting research • To disseminate the outcomes of HTA research, and follow-up/monitor impact.
*Health service* *managers*	• To understand implications of competing spending options and to manage resources accordingly • To create and manage local capacity for communications, knowledge translation and setting clinical standards.
*Courts and the* *judiciary*	• To understand the rationale for priority setting, and the tools and processes for evidence-informed priority-setting • To respect and rely on the government’s healthcare coverage choices where these have been made through evidence-informed priority-setting mechanisms in a procedurally legitimate manner as set out in law, while maintaining appropriate independence • To hold decision-makers accountable in the priority-setting process, for example through engaging in judicial review
*Patients and the* *public*	• To understand the implications of policy and clinical decisions, identify the extent to which they are evidence- informed and represent efficient and ethical use of public monies • To understand that unavoidable trade-offs have to be made in priority-setting and the associated ethical implications • To participate in the process of decision-making, recognising the need that decisions have to be made, and highlighting the extent to which they reflect societal values
*Producers of evidence*	
*Academic* *institutions,* *researchers and* *research managers*	• To understand policy and professionals decision-makers’ needs, • To identify those needs that can be satisfied by HTA research • To conduct and manage the required research without partisan advocacy and to the required standards • To communicate research effectively to meet the needs of decision-makers.
*Knowledge brokers*	
*Knowledge* *brokers, including* *priority-setting* *institutions*	• To understand the cultures of both research and decision-making environments • To assess and communicate research evidence and policy needs • To identify the ‘right’ stakeholders from both sides and to convene, facilitate and mediate between them such that there is meaningful knowledge transfer between researchers and decision makers (and between government agencies to local hospitals, professional organisations and community workers, and so on).
*Media* *organisations and* *journalists*	• To report in an objective and impartial manner stories linked to priority-setting in health and to institutions set up by governments to make such decisions • To encourage public debate in a positive way, and improve policymaking through holding decision makers accountable to the general public

HTA = health technology assessment; INNE = Individual, Node, Network, Environment

Exerting direct influence on the Environment may be difficult. Thus, most capacity-building activities will target specific stakeholders at the lower levels, as means of impacting on a broader friendlier Environment for evidence-informed priority setting. This especially applies when engaging with the media, professional organisations, and with funders and supra-governmental bodies, who are well positioned to influence the broader Environment. Capacity building activities for other stakeholders could operate at the Network level, for example through supportive conferences or regionally based researcher/policy-maker meetings ( Hofman *et al.*, 2015).

Depending on local needs, targeting certain groups such as agencies newly tasked with evidence-informed priority setting could be part of a strategy to support Nodes. Nodes include units that produce evidence to inform priority-setting (e.g. the HTA Committee and its secretariat in Indonesia responsible for generating HTA recommendations), and groups who demand evidence to inform priority-setting (e.g. policymakers in health ministries who will consider HTA recommendations in their decision-making), as well as the knowledge brokers at the interface between the two and with patients and the general public ( Lomas, 2007).

Finally, all capacity-building activities ultimately involve Individuals; the potential impact of empowering individuals to become champions and leaders within their respective organisations and networks should not be underestimated ( West *et al.*, 2015).

Each of these stakeholders need different levels of understanding and skills, beyond the purely technical, and will therefore need different methods of training including formal and informal approaches. Suitable training resources will also need to be arranged and, if necessary, created. New institutions may be needed and the existing ones need to be adapted, and capacities currently spread across poorly connected individuals or institutions within a given country or region need to be identified and consolidated, and brought together into the network. It is also important to stress that inadequate attention to the capacity needs of any one target stakeholder can easily undermine efforts to build priority setting mechanisms that function effectively at other levels (see Figure 1). This is the keystone of the INNE approach. However, capacity building should never be done in isolation, but rather be an ongoing interdisciplinary and multiprofessional process involving knowledge transfer and exchange between stakeholders.

To develop the capacities for any target stakeholder group in any particular context, it would be necessary to assess the following:

- Adequacy of existing capacity
- Capacities target stakeholders think are needed
- Key outcomes target stakeholders want to achieve from capacity building
- The best strategy needed to address these capacity gaps
- Practical constraints that have been identified, such as human resource pipeline issues.

Such a baseline assessment will help ensure that all capacity building activities are appropriately addressed.

## Unpacking capacity needs at each level of the INNE framework

### The Environment level

***Capacities of the health system.*** The capacity of a health care system to support priority-setting, and the associated capacities across the various levels of INNE, requires institutionalising priority-setting agencies at provincial, national and regional levels, ensuring that appropriate structures, processes and incentives are in place. There are several major examples of agencies responsible for setting health priorities in entire countries, or parts of them in the case of federal systems of governance ( Dittrich & Asifiri, 2016). However, the analytical advantages and weaknesses of the various models are only beginning to be exposed, and their sustainability has yet to be fully tested ( Dittrich & Asifiri, 2016). Prescriptive guidance would therefore be premature, and any useful guidance would unlikely be one-size-fits-all.

Nevertheless, factors that may support institutionalisation of explicit priority setting in the context of LMICs have recently been identified in a policy brief co-authored by members of HTA agencies belonging to HTAsiaLink, a regional network ( Chootipongchaivat *et al.*, 2016). Its recommendations (see Box 1) are based on the experience of seven settings: China, Taiwan, Indonesia, the Republic of Korea, Malaysia, Thailand and Vietnam. The authors identify five conducive factors for HTA development and provide a practical step-by-step guide, including a checklist for monitoring the progress of HTA introduction and development ( Chootipongchaivat *et al.*, 2016). Although the policy brief focuses on the use of HTA to inform coverage decisions under universal health coverage (UHC), these recommendations could also be applied to HTA in general resource allocation.

Box 1. **Recommendations for the development of HTAs, adapted from the policy brief *Conducive Factors to the Development of Health Technology Assessment in Asia* ( Chootipongchaivat *et al.*, 2016)**

1. **Human resource development** within HTA research organizations as well as decision-making bodies and other relevant stakeholders using HTA.
2. **Development of core team or HTA institutes** committed to HTA who will coordinate HTA activities and gain the trust of partners
3. **Linking HTA to policy decision-making mechanisms** including the pharmaceutical reimbursement list/essential drug lists, immunization programs, high-cost medical devices package, and public health programmes.
4. **Implementing HTA legislation** to ensure sustainability through participation, transparency, and systematic application of HTA in the policy process rather than focusing on technical issues.
5. **International collaboration,** especially in the formative stages, for financial and technical capacity building support and sustained international knowledge exchange across agencies in the longer term.

Any priority-setting frameworks that simply generate evidence of what works and represents good value for money are inadequate ( Chootipongchaivat *et al.*, 2016; Rutter, 2012). “Good value” policy options may have no direct bearing on financial protection, and the distribution of financial and disease burden, which are important issues for UHC ( Voorhoeve *et al.*, 2016). In addition, the “right” decisions, even when evidence-based, don’t always get implemented for a number of practical, political or other reasons. It may be entirely rational for policymakers to make decisions against evidence-based recommendations, if by that they suit their own political interests, for instance to win electoral support from the ‘median voter’ (whose particular concerns may differ from what would benefit the population on a whole) ( Hauck & Smith, 2015). This underscores the importance of developing a robust, principled process that considers such constraints, within which explicit methods for evidence-informed priority-setting can be institutionalised ( Chalkidou *et al.*, 2016b).

When we refer to ‘institutionalising’ priority-setting and HTAs, we seek to emphasise the importance of developing accepted norms and rules, and sustaining effective working relationships between relevant policymakers and research institutions ( Hawkins & Parkhurst, 2016; March & Olsen, 2008). Norms and rules based around notions of transparency, accountability, citizen engagement, openness, deliberation, and contestability are valuable beyond having intrinsic moral merits, because they improve both the quality and credibility of decisions arising from evidence-informed priority-setting ( Culyer, 2012; Daniels, 2000). Relevant processes that should be built in when institutionalising priority-setting and HTA include ( Culyer, 2012; Daniels, 2000):

- The possibility of external comment so that interested parties may see what there is to comment on;
- Consultation, through which external parties are invited both to engage with decision makers and their advisers and to enter into discussion about whatever aspects of the process may be underway at the time. These include assumptions, comparators, model building, literature review, and matters to do with the process itself;
- Appraisal of evidence, including evidence about publicly held values, evidence brought to the deliberation process by clinical and other professional participants, and discussions on how best to proceed when evidence is poor, second hand, irrelevant (as may be the case with evidence from high-income settings that is being considered in a LMIC context), or completely absent;
- Deliberation, the most complete form of engagement, in which relevant stakeholders participate in the actual decision making themselves. The final determination or conclusion of the process may be excluded from this process, since that responsibility most likely lies with those having political accountability.

These processes contribute to good governance in evidence-informed priority-setting, which enables it to become more resilient to vested interests and political change ( Hawkins & Parkhurst, 2016).

### At the Network level

***Capacities of funders and development partners.*** Global funders and development partners, including supra-governmental organisations like the World Health Organisation (WHO) and the World Bank, have significant power in shaping health priorities at the country level in LMICs (especially in low-income countries) ( Chalkidou *et al.*, 2016b; Glassman & Chalkidou, 2012). This can operate directly through their purchasing or provision of specific health care interventions, delivery platforms, and investment into research and technical assistance activities related to the above; or indirectly through their role as setters of global standards and norms, for example with the iDSI Reference Case ( Wilkinson *et al.*, 2016) and WHO CHOICE ( Chalkidou *et al.*, 2016b).

Funders and development partners need the specific capacity to commission, receive, interpret and use HTA and priority-setting research to inform not only their own choices in global health, but also the global standards and norms which client countries look to. Health system strengthening efforts could also be targeted towards the multitude of stakeholders and capacity gaps identified here, with the broader objective of supporting effective, evidence-informed and sustainable priority-setting that is country-owned ( Chalkidou *et al.*, 2016b). There should be a shared understanding within and between funders, delivery partners and LMIC country partners of the goals or outcomes of aid investment, in terms of funding, research outputs and technical assistance. This shared understanding could take the form of a common theory of change, that is a framework outlining the preconditions, causal linkages and assumptions underlying the desired investment goals ( Li, 2016).

At an internal iDSI Board meeting in Bangkok in January 2016, we asked four funder representatives who were present (from the Bill and Melinda Gates Foundation, UK Department for International Development, Rockefeller Foundation and the World Bank, respectively) what internal capacity-building they felt would be useful in their organisations in order to support priority-setting better. Three funders felt that their organisations should develop rapid response services for country partners requesting technical assistance, both in terms of being able to direct them to relevant and useful evidence sources as well as identifying international experts capable of providing immediate short-term technical support. The fourth funder reiterated the importance of having the capacity to use value for money in guiding investment decisions, pointing to the iDSI Reference Case ( Wilkinson *et al.*, 2016) and other ongoing efforts to incorporate components of HTA in the grant-making process.

### The Node and Individual levels: Consumers of evidence

***Capacities of policy and professional decision makers.*** There seems to be considerable variation in the extent to which both policy and professional groups possess the capacities detailed in Table 1, and there has been little research thus far that documents it (though see Hailey & Juzwishin, 2006, highlighting that the inability of policymakers to formulate appropriate questions risks diminishing the policy-relevance of HTA programmes). Routine follow-up and monitoring of impact of HTA research by decision makers as an integral part of evidence-informed priority-setting is rare, as is evidence of any matching training programmes targeted at developing such capacities among policymakers. Fundamentally, there needs to be political commitment among policy leaders to progress to UHC and use evidence and tools such as HTA to help achieve that aim ( Li *et al.*, 2016).

***Capacities of health service managers.*** NICE in the UK engages service managers in their HTA processes to select healthcare interventions and clinical guideline recommendations at the national level ( National Institute for Health and Care Excellence, 2015). Among clinical research and health services research in general, however, health service managers are rarely included. This is possibly both symptomatic of and perpetuating the phenomenon that most HTA has focused on comparing individual interventions, as opposed to service delivery platforms or different organisational modes for human resources ( Morton *et al.*, 2016). HTA may therefore not provide sufficient information on the broader financial and organisational implications of competing resource allocation strategies that health service managers need in order to make fully informed decisions ( MacDonald *et al.*, 2008). The use of HTA to support planning by local health service managers in the UK has also arguably been hindered because of the relative inaccessibility of, and concerns over the acceptability of, the specialist methods employed ( Airoldi *et al.*, 2014).

Irrespective of the scope and complexity of HTA, the practical implementation of evidence-informed policies and practices crucially depends the on managers’ ability to set and enforce clinical standards and gain local adoption of good practice in both primary and secondary care settings, arranging funding and bringing local communities along through supportive and constructive local engagement. In the UK the National Health Service (NHS) has a relatively well-established tradition of clinical governance ( Scally & Donaldson, 1998; Swage, 2003), and routine performance measures of healthcare managers and providers now include how successful they are in implementing clinical governance ( National Institute for Health Research, 2017). In LMICs on the road to UHC, the capacity of health service managers to understand the implications of evidence-informed developments, competing spending options, and of managing resources accordingly will require specific training and ongoing support.

***Capacities of patients and the public.*** Setting priorities in health implies that some interventions and some patient groups will be covered and others will not. There is a risk that because of this, those who do not see themselves as privileged, along with their carers and supporters, lose whatever enthusiasm they may have had for developing UHC. Their continuing engagement, and understanding of the process and decisions, are important both morally and for the success of the strategy ( Clark & Weale, 2012).

Patients and the general public need to understand the implications of policy and clinical decisions and of the decision-making process, the extent to which specific decisions are evidence-informed and represent efficient and ethical use of public or private money, and they need to participate with an active voice in decision shaping that affects their interests. Capacity development activities could include training of health workers, patients and the public in research projects in the field, and other forms of patient involvement (e.g. HTA appraisal panels, citizens’ juries) ( Littlejohns & Rawlins, 2009) in conjunction with the development of tools to facilitate stakeholder engagement in priority-setting ( Bolsewicz Alderman *et al.*, 2013; Makundi *et al.*, 2007; Weale *et al.*, 2016). Any such tools will be context-sensitive, if not context-specific, and take into account the socio-cultural values and political environment of the country or region ( Bolsewicz Alderman *et al.*, 2013).

### The Node and Individual levels: Producers of evidence

***Capacities of academic institutions, researchers and research managers.*** Healthcare researchers for LMICs tend to regard research capacity development in terms of the acquisition of research skills (e.g. World Health Organization, 2015), mainly through masters and PhD programmes offered by major centres in high-income countries. They measure success in terms of the various kinds of training received and in authorship in so-called ‘high-impact’ journals, which are predominantly published in English. Equally important, however, is how skilled local research communities are in engaging with policy and professional end-users, discerning their decision-related needs for evidence, and identifying what is researchable, and in translating those needs into research projects and programmes that can be implemented locally (with or without assistance from elsewhere). For low-income countries the key lessons to be learned may lie not with high-income countries, but with middle-income countries.

Networks that link the research community to policy decision-makers, professional regulators and professional colleges ( Ezeh *et al.*, 2010), as well as institutional and personal relationships, all need to be explored further. These relationships exist to some extent in all countries but may not focus particularly on the development of strategic commitments to the provision of timely and relevant evidence and analysis, or their institutionalisation into established practices through standing committees, routine communication (e.g. electronic) and other standard operating procedures ( Hawkins & Parkhurst, 2016; March & Olsen, 2008).

With respect to technical capacities for research in LMICs, while there are relatively abundant resources in public health and epidemiology (although Ezeh *et al.*, 2010, highlighted particular gaps in Sub-Saharan Africa), there is an even greater shortage of skills in high quality economic evaluation that would enable research teams to offer evidence of cost-effectiveness to achieve better and more equitably distributed health outcomes ( Doherty, 2015; World Health Organization, 2015). In Sub-Saharan Africa, there is also a shortage of competency in systematic reviewing, especially in reviewing designed to economise on the need for new research by appropriate and critical translation of results from previous studies ( Doherty, 2015). There also exists limited networks between African institutions in terms of research collaborations in health economic evaluation; the collaborations that do exist tend to be with North American or European institutions ( Doherty, 2015; Hernandez-Villafuerte *et al.*, 2016). The significant health economic research activity, capacity, and capacity-building initiatives in related disciplines that already exist in South Africa suggest that it is well placed as a hub country for catalysing South-South collaborations with other African countries ( Ezeh *et al.*, 2010; Hernandez-Villafuerte *et al.*, 2016).

A more comprehensive and strategic approach to capacity building might embody the following additional features (see also Ezeh *et al.*, 2010):

*Leadership, management and administration*

- A general commitment to creating training opportunities for research managers and trainers of technical, management and leadership skills in research, and developing local centres of excellence without creating lasting dependencies on foreign centres of excellence;
- A formal system for identifying local training needs for multi-disciplinary and professional competencies and the recipients of training, with a particular focus on South-South engagements
- A formal system for training in skills required for middle and senior research managers
- A comprehensive attempt to match training courses of all kinds (full time, part time, short, long, with or without internships, in workplaces or at special centres, with a range of certificated competence or one, etc.) and for various purposes (single discipline, exposure to cognate or complementary disciplines);
- Training in research grant application and management
- Participation in a strategy for increasing the ability of universities and research centres (public and private) to train junior researchers and take on leadership roles.

*Technical and research skills*

- A strategic assessment of the multi-disciplinary skills required in each context, including professional skills in economic evaluation and application of the iDSI Reference Case, and consideration of equity and other ethical objectives where relevant ( Norheim, 2016)
- Recruitment of researchers into disciplines where more skilled workforce is needed
- Training in interpretation of transferability (sometimes termed generalisability) of research evidence developed elsewhere than in the country of potential application
- Training in systematic reviewing.

*Knowledge transfer and exchange*

- Training in knowledge transfer and exchange (other than communication to fellow academics) to ensure that research is timely, understandable and useful for the target audience. This involves engaging decision-makers in research processes, synthesising interdisciplinary knowledge into key actionable messages for relevant decision-makers, and disseminating plain language research summaries via a range of channels other than academic publications, including social media and face-to-face exchanges between researchers and end-users ( Lavis, 2016; Lomas, 2007; see also section on Capacities of knowledge brokers)
- Training in fit-for-purpose publication plans with specific readerships in mind
- Non-self-serving clarity as to the meaning of “high quality” research and “high quality” research outlets. What this refers to is research that is rigorously conducted and reported, genuinely novel, and relevant to policy and clinical practice, and research outlets which have transparent, rigorous editorial and peer-review policies, and are trusted by and influential among academic and policy leaders in the given field, but not necessarily restricted to so-called ‘high impact’ journals in English.

### The Node and Individual levels: Knowledge brokers

***Capacities of knowledge brokers.*** Knowledge brokers and knowledge brokering agencies are intermediaries between worlds of research and action ( Lomas, 2007). Their role involves “all the activity that links decision makers with researchers, facilitating their interaction so that they are able to better understand each other’s goals and professional cultures, influence each other’s work, forge new partnerships, and promote the use of research-based evidence in decision-making.” ( Canadian Health Services Research Foundation, 2003). Capacity building is part of their philosophy: for researchers to be able to do applied research and decision-makers to be able to use it ( Lomas, 2007).

Knowledge brokers can push for improvements on the evidence-supply side, for instance by packaging it better and by disseminating it in a more organised way ( Lavis, 2016). They can also work on the evidence-demand side, for instance by advocating for the creation of institutional mechanisms that privilege the use of research evidence and building capacity to find and use research evidence efficiently ( Chalkidou *et al.*, 2016b; Lavis, 2016).

To achieve all of this, knowledge brokers must have the capacity to understand the cultures of both the research and decision-making environments. They need to be able to identify the ‘right’ stakeholders from both sides, and achieve meaningful knowledge transfer between them. Stakeholders from the different environments include researchers and decision makers, government agencies and local hospitals, professional organisations and community workers, and so on.

Of particular relevance to LMICs, where capacities on both demand- and supply-sides may be sparse, is focusing capacity-building efforts on existing agencies or groups of individuals with some formal linkage between the research and decision-making circles, including those who themselves function as a research unit (for example, the technical unit within a ministry of health) ( Li, 2016). HITAP in Thailand is a good example of an institution with dual function as a generator of primary research in health economics and health policy, and as a knowledge broker through HTA processes that convene stakeholders including policymakers, clinicians, and civil society ( Jongudomsuk *et al.*, 2012). **

***Capacities of media organisations and journalists.*** The ongoing claims of a finite budget made by different stakeholders lie at the crux of priority-setting in health, and in many countries the media wield significant power to influence how these claims are understood by the general public and acted upon by policymakers, an issue perhaps more important than ever in the so-called “post truth” era ( Marmot, 2017). We mean media in the broadest sense, so we are including journalists and editors in TV and print and those who communicate primarily through electronic media such as Twitter and blogs, in particular those with a specialist interest in health, government policy, science, or development.

While the role of the media varies from country to country, there will be technical, political and ethical issues in priority-setting that are shared across settings ( Briggs, 2016; Hauck & Smith, 2015; Kieslich *et al.*, 2016; Rumbold *et al.*, 2017). There will also be general principles and common challenges to overcome in understanding and communicating notions such as priority-setting, rationing and fair access to services, for example the fact that evidence-informed priority-setting decisions are made with the whole population in mind but will inevitably lead to winners and losers among individual patients. We are not suggesting any compromise to editorial independence or the need for journalists to hold key stakeholders accountable. Instead, the aim is to encourage a greater understanding of the complexity of the priority setting process and to enable better informed and impartial reporting.

## Discussion

Setting explicit priorities in health is not simply a narrow technical exercise. It involves the mobilisation of a wide range of skills and experience. There are many types of “capacity” required – not only the capacity to “do” research. If the aim is to get research translated into policy, in a procedurally legitimate manner, a strategy for capacity building needs to take into account the various stakeholders involved in the evidence-to-policy continuum.

We have outlined the kinds of capacity needed to support decision makers when setting health priorities, where such capacity can be found, and how it can best be created. We have set out a framework for understanding the key elements of capacity building, and how iDSI partners are currently involved in supporting capacity development. Application of the INNE framework highlights the broad range of stakeholder groups that need to be targeted in capacity building when setting health priorities, particularly in LMICs. It follows therefore, that there is no single approach to capacity building, but rather a spectrum of activities that recognise the different roles and skill sets of all those involved in the process. It will require dedicated resources, and nurturing of traditional academic expertise will be one of many important components.

### Recommendations for further research

In Table 2 we propose a set of research recommendations addressing the capacity needs of different stakeholder groups in priority-setting, in order to inform any future capacity building strategy adopted by iDSI or other development initiatives. Given the focus on targeting different stakeholders, we also recommend that a tool for mapping relevant stakeholder groups be developed that can adapt to different national contexts ( Li, 2016).

**Table 2.** Research recommendations to address capacity needs for priority-setting, including understanding the capacities of different stakeholders in specific countries and tools to help capacity-building.

Stakeholder group	Research recommendations
*Environment*	
*Health system*	• Further detailed review of established priority-setting agencies including those in Australia, Korea, Thailand, Malaysia, Taiwan, Canada, the UK explaining their roles in the particular contexts for which they were developed and noting the characteristics that might be most adaptable to conditions in LMICs.
*Networks*	
*Funders and* *development* *partners*	• Develop, implement and evaluate common theories of change and indicators around priority-setting in health, so that investment efforts (in terms of funding, research and technical assistance) can be consolidated and strategically driven towards common outcomes. • Methodological specification as well as implementation of value for money principles, such as those espoused in the iDSI Reference Case, as well as ongoing reflection on the part of funders about their own capacity development needs, will help to accelerate the process.
*Nodes (organisations) and Individuals*	
*Consumers of evidence*	
*Policy and* *professional* *decision makers*	• Survey the capacities in policy and professional circles in LMICs (for instance in the iDSI collaborating countries: China, India, Indonesia, South Africa, Vietnam), and identify the training that exists for leaders in those fields. The intention will be to identify and share good practice from which all may learn and which might provide an agenda for more detailed work on the effectiveness and cost-effectiveness of the various interventions aimed at increasing decision-makers’ capacities to commission, use and monitor research.
*Health service* *managers*	• Review the existing training and support arrangements for health service managers and explore with selected groups the most cost-effective ways of meeting their needs in particular contexts, identifying appropriate syllabuses and methods of delivery through graduate training and continuing professional development courses and workshops. • Understand, at the methodological and policy levels, how concepts and methods of priority-setting and health technology assessment could be practically applied to resource allocation problems beyond individual healthcare interventions, and more broadly to healthcare delivery platforms and human resource issues.
*Patients and the* *public*	• Develop tools and approaches that will support decision-makers in identifying the purposes of their patient and public engagement strategies, and test out such tools and approaches in LMIC settings. The aim is to increase the likelihood that engagement strategies will be aligned with policy goals, support inclusion and representation of key stakeholders affected by priority-setting decisions. By facilitating inclusion of locally specific ethical considerations into priority-setting, and protect engagement activities from common pitfalls, this could ultimately improve decision-making and enable it to be more effective and fair.
*Producers of evidence*	
*Academic* *institutions,* *researchers and* *research managers*	• Develop a handbook of best practices for understanding the needs of policy and professional decision-makers; identifying the extent to which such best practices are context-dependent, and the means of sharing them between policy, professional and research partners. The research will be qualitative and descriptive, embody both recommended principles and practical examples drawn from extensive consultation from both researchers and end users, and provide an agenda for more detailed work on the effectiveness, cost-effectiveness and fairness of the various ways of communication and knowledge translation.
*Knowledge brokers*	
*Knowledge* *brokers, including* *priority-setting* *institutions*	• Identify knowledge brokers in countries, using tools such as social network analysis (Shearer *et al.* 2014), with the goal of influencing the key players who are strategically best placed to facilitate evidence-informed priority- setting. • Support the development of knowledge brokers’ technical and institutional capacities, including the capacity to convene and hand-hold other evidence generators together with evidence users (decision-makers).
*Media* *organisations and* *journalists*	• Through workshops and other platforms, convene journalists and editors to share and establish best practices for objective and impartial reporting of stories linked to priority-setting in health and to institutions set up by governments to make such decisions, as a means of informing and influencing the other stakeholder groups (including policymakers and the general public) • Understand and develop existing efforts for the networking and capacity-building of relevant journalists.

iDSI = International Decision Support Initiative; LMIC = low- and middle-income country

Capacity building should be a two-way process; those who engage in capacity building should also reflect on their own capacity development to ensure their activities have the impact desired in the short and long term ( Itad & NICE International, 2016). iDSI has a Monitoring, Evaluation and Learning framework to track ongoing implementation, collect evidence of iDSI contributions to stated aims, enhance accountability to members, stakeholders and funders, and encourage ongoing reflection and learning ( Li, 2016). In addition, iDSI and its core partners have subjected themselves to independent reviews in order to reflect on progress, achievements, and operational arrangements ( Health Intervention and Technology Assessment Program, 2009; Itad & NICE International, 2016). A Mid-Term Learning Review has been conducted to ensure iDSI remains fit-for-purpose and help identify potential capacity gaps and how these can be addressed (international Decision Support Initiative, in preparation).
