# Supplementary material for: Evidence-informed capacity building for setting health priorities in low- and middle-income countries: A framework and recommendations for further research
Source: F1000Res. 2017 Mar 7;6:231. [Version 1] doi: 10.12688/f1000research.10966.1 (PMC5497935; doi:10.12688/f1000research.10966.1)
Supplement: Peer review history

# Peer review: Referee response for version 1

**DOI:** 10.5256/f1000research.11820.r24030

**Published:** 2017-07-04

**Authors:** Lilian Dudley

**Affiliations:** 1 Division of Community Health, Department of Interdisciplinary Health Sciences (DIHS), Stellenbosch University, Stellenbosch, South Africa

The article discusses the important topic of capacity building for priority setting in health in LMIC’s, by a research collaboration which draws on broad experience and expertise. The article reviews current knowledge on the topic, uses a framework to organise capacity building for different levels and stakeholders, and provides a useful way of documenting the wide range of groups and activities that are required to strengthen capacity for priority setting in health.

 Comments:-

1. The title is a bit misleading as it suggests that the topic is about evidence informed approaches to capacity building, but then presents very limited evidence of approaches to capacity building for priority setting. Perhaps the title should be ‘Capacity building for evidence informed health priority setting in LMIC’s?
2. The scope of priority setting in health in the context of this article is not clearly defined. The article starts and ends by broadly discussing priority setting in health in LMIC’s, and links this loosely to Universal Health Coverage (UHC). The body of the article and the framework however focuses almost entirely on capacity building for Health Technology Assessment. It is therefore not clear whether the intention was to review capacity building needs for HTA specifically or more broadly for priority setting for programmes and other activities in the health sector. It would be useful to clarify this for readers, as HTA does require a specific set of technical skills and understanding both by producers and users of the knowledge, which may differ from other forms of priority setting.
3. Capacity building is discussed broadly as a process, and the article focuses on stakeholder mapping to identify the levels and groups that require capacity building. The ‘capacities required’ again focuses more on HTA as a ‘content’ area, than a broad approach to priority setting in health, for which a more comprehensive approach would be required to include assessment of need, societal preferences, participatory processes etc.
4. Very little is said about capacity for good governance and leadership in priority setting, and ways in which these should be strengthened as a critical component of improving priority setting in health systems in LMIC’s. It would be useful to reflect on these and how the proposed framework could include these components of the health system.

I have read this submission. I believe that I have an appropriate level of expertise to confirm that it is of an acceptable scientific standard.

# Peer review: Referee response for version 1

**DOI:** 10.5256/f1000research.11820.r21566

**Published:** 2017-04-19

**Authors:** Clara Richards

**Affiliations:** 1 International Network for the Availability of Scientific Publications (INASP), Oxford, UK

I think the article takes a comprehensive approach to capacity building, its importance, opportunities and how to conduct it. However, I would suggest to include a definition of capacity development - it is not very clear how the authors understand capacity at the environmental level for example. 

 I think that the frameworks that the authors refer to are good however I would suggest using those that take a complexity lens as well, since the problem that is discussed is complex and involves multiple stakeholders. I recommend taking a look at the ITAD framework http://www.itad.com/knowledge-and-resources/capacity-development-2/ or an even more comprehensive one 'Context Matters' http://www.politicsandideas.org/contextmatters/

 Related to the above I feel that the article could take a systems approach that takes complexity into account. What does it take to change and improve systems? Usually, the problem lies beyond technical capacity but it is more about understanding and sharing a common problem, being able to identify and relate to different points of views and realities, ability to find solutions jointly, an ability to work with different parts of the system, etc. Some of these are addressed in the article (like leadership skills) but drawing on further systemic thinking could help (see Ken Wilber).

I have read this submission. I believe that I have an appropriate level of expertise to confirm that it is of an acceptable scientific standard.
